# Exploring barriers and facilitators to integrated hypertension-HIV management in Ugandan HIV clinics using the Consolidated Framework for Implementation Research (CFIR)

**DOI:** 10.1186/s43058-020-00033-5

**Published:** 2020-05-04

**Authors:** Martin Muddu, Andrew K. Tusubira, Brenda Nakirya, Rita Nalwoga, Fred C. Semitala, Ann R. Akiteng, Jeremy I. Schwartz, Isaac Ssinabulya

**Affiliations:** 1 Department of Internal Medicine, Makerere University College of Health Sciences, Kampala, Uganda; 2 Uganda Initiative for Integrated Management of Non-Communicable Diseases (UINCD), Kampala, Uganda; 3 Makerere University Joint AIDS Program (MJAP), P.O. Box 7587, Kampala, Uganda; 4 Section of General Internal Medicine, Yale School of Medicine, 333 Cedar Street, New Haven, CT 06511 USA; 5 Uganda Heart Institute, Mulago Hospital Complex, Kampala, Uganda

**Keywords:** Barriers, Facilitators, Hypertension and HIV integration, Uganda, CFIR

## Abstract

**Background:**

Persons living with HIV (PLHIV) receiving antiretroviral therapy have increased risk of cardiovascular disease (CVD). Integration of services for hypertension (HTN), the primary CVD risk factor, into HIV clinics is recommended in Uganda. Our prior work demonstrated multiple gaps in implementation of integrated HTN care along the HIV treatment cascade. In this study, we sought to explore barriers to and facilitators of integrating HTN screening and treatment into HIV clinics in Eastern Uganda.

**Methods:**

We conducted a qualitative study at three HIV clinics with low, intermediate, and high HTN care cascade performance, which we classified based on our prior work. Guided by the Consolidated Framework for Implementation Research (CFIR), we conducted semi-structured interviews and focus group discussions with health services managers, healthcare providers, and hypertensive PLHIV (*n* = 83). Interviews were transcribed verbatim. Three qualitative researchers used the deductive (CFIR-driven) method to develop relevant codes and themes. Ratings were performed to determine valence and strengths of each CFIR construct regarding influencing HTN/HIV integration.

**Results:**

Barriers to HTN/HIV integration arose from six CFIR constructs: organizational incentives and rewards, available resources, access to knowledge and information, knowledge and beliefs about the intervention, self-efficacy, and planning. The barriers include lack of functional BP machines, inadequate supply of anti-hypertensive medicines, additional workload to providers for HTN services, PLHIV’s inadequate knowledge about HTN care, sub-optimal knowledge, skills and self-efficacy of healthcare providers to screen and treat HTN, and inadequate planning for integrated HTN/HIV services.

Relative advantage of offering HTN and HIV services in a one-stop centre, simplicity (non-complex nature) of HTN/HIV integrated care, adaptability, and compatibility of HTN care with existing HIV services are the facilitators for HTN/HIV integration. The remaining CFIR constructs were non-significant regarding influencing HTN/HIV integration.

**Conclusion:**

Using the CFIR, we have shown that while there are modifiable barriers to HTN/HIV integration, HTN/HIV integration is of interest to patients, healthcare providers, and managers. Improving access to HTN care among PLHIV will require overcoming barriers and capitalizing on facilitators using a health system strengthening approach. These findings are a springboard for designing contextually appropriate interventions for HTN/HIV integration in low- and middle-income countries.

Contributions to the literature

- We used the widely used and validated CFIR to assess the HIV program for HTN/HIV integration.
- To our knowledge, this is the first study to explore barriers and facilitators to integrating hypertension screening and treatment into HIV clinics using the CFIR.
- The barriers and facilitators identified are a basis for designing contextualized implementation interventions for HTN/HIV integration in Uganda and other LMIC using a health system strengthening approach.

## Background

Persons living with HIV (PLHIV) and receiving antiretroviral therapy (ART) are at increased risk of cardiovascular disease (CVD) [1–3]. In Uganda, approximately 1/3 of PLHIV aged ≥ 18 years have hypertension (HTN), the leading cause of CVD, and preventable mortality [4–12]. PLHIV with HTN have an increased risk of mortality compared to HIV negative persons [13].

The World Health Organization (WHO) and Uganda Ministry of Health (MoH) consolidated guidelines for HIV care and treatment and recommend that all PLHIV should be screened for HTN at every visit to the HIV clinic. PLHIV who are diagnosed with HTN should receive treatment for both HIV and HTN as integrated services [14, 15]. HTN/HIV integration provides patient-centred care compared with vertical programs and increases efficiency, by eliminating fragmentation and duplication of services [16]. However, there is little empirical evidence for HTN/HIV integrated services in Uganda.

HTN/HIV integration has been attempted in Uganda by the SEARCH trial. However, although HTN screening was achieved for all patients in the HIV clinics under this program, control of HTN among PLHIV who received antihypertensive medication remained suboptimal at about 30% [17].

We recently conducted a retrospective cohort study of 1649 PLHIV. We mapped the parallel care cascades for HTN and HIV within three high volume HIV clinics (average 3600 PLHIV) in Eastern Uganda. In these HIV clinics, we demonstrated suboptimal HTN screening, one year retention in HTN care, and HTN control of 27%, 57%, and 24%, respectively, among PLHIV within a successful HIV program that has achieved the two of three UNAIDS 90-90-90 goals [18]. As a follow-up to that study, this qualitative study sought to determine barriers to, and facilitators of, integrating screening and treatment of HTN into HIV clinics in Eastern Uganda. Understanding the barriers and facilitators would inform the design of contextually appropriate implementation interventions for HTN/HIV integration in Uganda.

## Methods

### Study design

Using data from our previous retrospective cohort study, we graded HTN care cascade performance among the three HIV clinics as high, intermediate, and low according to achievement in screening, diagnosis, initiation of treatment, retention, monitoring, and control.

We conducted key informant interviews (KIIs) with the district health officer (DHO) and healthcare providers at the three HIV clinics. Additionally, to obtain patients’ information about integrated HTN/HIV services, we conducted focus group discussions (FGDs) and in-depth interviews (IDIs) with hypertensive PLHIV at each HIV clinic.

**Fig. 1:**
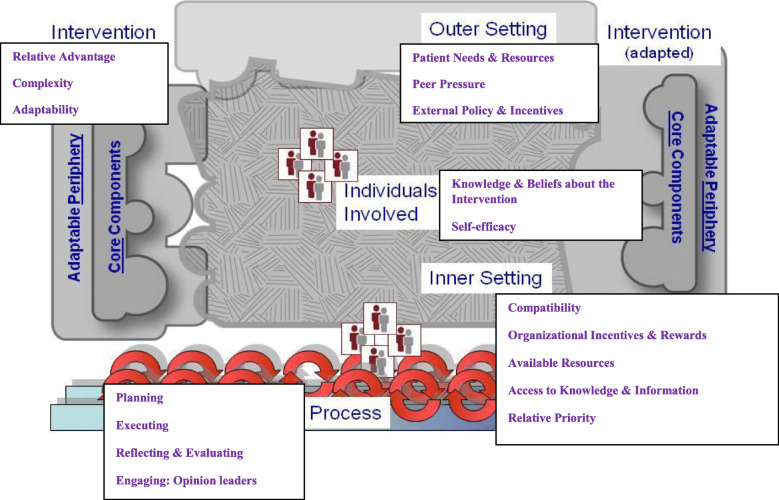
CFIR domains and constructs which emerged in the study (Damschroder et al. [19])

We utilized the Consolidated Framework for Implementation Research (CFIR) to explore barriers to and facilitators of HTN/HIV integration [19]. We chose the CFIR because it provides a pragmatic structure for identifying potential influences on implementation of interventions in health systems at multiple levels [19, 20]. CFIR is preferred for this study since we are exploring barriers and facilitators, at multiple levels of the healthcare system, for integrated HTN/HIV care. CFIR organizes conceptual elements across theories and disciplines into 39 constructs which are then organized in five key domains. All constructs interact to affect the process and effectiveness of implementation [21]. CFIR’s five major domains include intervention characteristics, outer setting, inner setting, characteristics of individuals, and implementation process [22] (Fig. 1). We evaluated all the 39 CFIR constructs. However, we report on the constructs which emerged from the interviews and FGDs. Barriers to and facilitators of HTN/HIV integration (Intervention) were compared across the three HIV clinics to understand the strength of their influence on HTN screening and treatment among PLHIV.

### Study setting

We conducted this study at three HIV clinics (The AIDS Support Organization (TASO) Tororo, Nagongera Health Centre IV, and Mulanda Health Center IV) and the District Health Office of Tororo District, Eastern Uganda.

The Ugandan public-sector healthcare system is hierarchical in nature and comprises the national (Ministry of Health [MoH]), sub-national (regional), district, health facility, and community levels. MoH develops guidelines for health services and rolls them out through the regions to the districts. The District Health Team (DHT) leads and coordinates guideline implementation at health facilities.

HIV clinics are the designated treatment centers for HIV, HIV-associated opportunistic infections, and other HIV-associated co-morbidities. They are physically situated as outpatient departments within health centers and hospitals. We selected these three HIV clinics because they are the largest in Tororo district, providing care to approximately 7500, 1400, and 1100 PLHIV, respectively. They are housed within public health facilities with support from both the Government of Uganda and the President’s Emergency Fund for AIDS Relief (PEPFAR). The clinics are staffed by various cadres of health workers including clinicians, nurses, midwives, and HIV peer counselors. Each HIV clinic offers a full spectrum of HIV services including screening, ART, viral load testing, and screening for and treating opportunistic infections. Each clinic also has the mandate to screen for and manage NCDs such as HTN and diabetes. Based on existing processes in place at the time of data collection, within a given clinical encounter, blood pressure (BP) is measured by the clinician at his/her discretion [18]. If a patient is diagnosed with HTN (by measurement or previous history), the clinician typically prescribes both ART and antihypertensive medicine simultaneously, and the client is given one follow-up appointment for both conditions. All medicines at facility pharmacies are obtained from the centralized National Medical Stores (NMS). PEPFAR provides funds to NMS to procure medicines specifically for HIV and opportunistic infections. Medicines for HTN and other non-communicable diseases (NCDs) are procured on request by the health facilities via general funds allocated to each health facility by MoH. If medicines are out of stock at the facility pharmacy, the patient is advised to purchase them from a private sector pharmacy of the patient’s choice.

Clinic providers are routinely oriented to national HIV treatment guidelines which recommend screening for NCDs and their risk factors. In addition, clinical support discussions about challenging HIV/NCD cases were also used to build capacity for NCD/HIV integration among clinicians.

### Study participants and sampling

We interviewed purposively selected healthcare providers and patients living with both HIV and HTN. Eligible healthcare providers were individuals who had responsibility to treat patients in the HIV clinics or leadership roles at the respective health facilities or at the district health office. These healthcare providers were knowledgeable about and actively involved in HIV service delivery at their clinics or at the district health office. Eligible patients were PLHIV with hypertension attending one of the three HIV clinics. Patients with a mental disability were excluded. We approached patients through telephone calls and healthcare providers face to face. All participants approached consented to participate. We recruited participants until we achieved data saturation.

### Data collection

We used semi-structured interview guides developed based on the five domains of CFIR [19]. The interviews had open ended questions reflecting patient, provider and healthcare managers’ perspectives, and perceptions about HTN/HIV integration. Prior to data collection, we pretested the interview guides with healthcare providers and hypertensive PLHIV at TASO Tororo who were not participating in the study. AKT, DBN, and RN shared the objectives of the study with the DHO and healthcare providers and conducted the FGDs and IDIs. AKT is a male public health specialist while DBN and RN are female social scientists with expertise in public health. All three authors who conducted interviews were not part of the healthcare team at the HIV clinics but had experience in conducting FGDs and IDIs. The interviewers established a relationship with the DHO and healthcare providers prior to study commencement. The interviewers had no bias or personal interest in the research. We conducted six FGDs (two per HIV clinic) with patients in each group consisting of ten hypertensive PLHIV lasting 60 min. We conducted twelve IDIs with hypertensive PLHIV, four at each HIV clinic, and eleven KIIs each lasting 30 min. We did not conduct any repeat interviews. All KIIs were conducted in English while IDIs and FGDs were conducted in Ateso and Japadhola, the local languages. We used patient IDIs to obtain individual lived experiences, while FGDs explored shared experiences among hypertensive PLHIV. The FGDs and interviews were conducted in a private space within the HIV clinics. All interviews were audio-recorded and transcribed verbatim. Transcripts in Ateso and Japadhola were translated into English. Participants were not engaged in reviewing transcripts.

### Data analysis

#### Qualitative data coding

After transcribing, a research team with expertise in social sciences, public health, and clinical care was established comprising three members (AKT, DBN, RN), who conducted thematic content analysis. The team coded transcripts using the deductive (guided by CFIR as a coding framework) approach. The coding process was guided by the consensual qualitative research (CQR) procedure [23].

First, each research team member reads three transcripts independently and identified preliminary codes. Through a series of meetings, discussing coding differences, an initial codebook was agreed upon. To organize and manage the large amount of data, all transcripts were then coded utilizing the Atlas.ti (version 7) software while applying the codebook and giving an allowance for new codes. An external researcher independently coded six of the transcripts to establish inter coder reliability (Kappa 0.80). A final codebook and subthemes were resolved by the researchers through more meetings, and these were mapped to the CFIR domains and constructs (Table 3).

### Defining unit of analysis and performance criteria

**Fig. 2:**
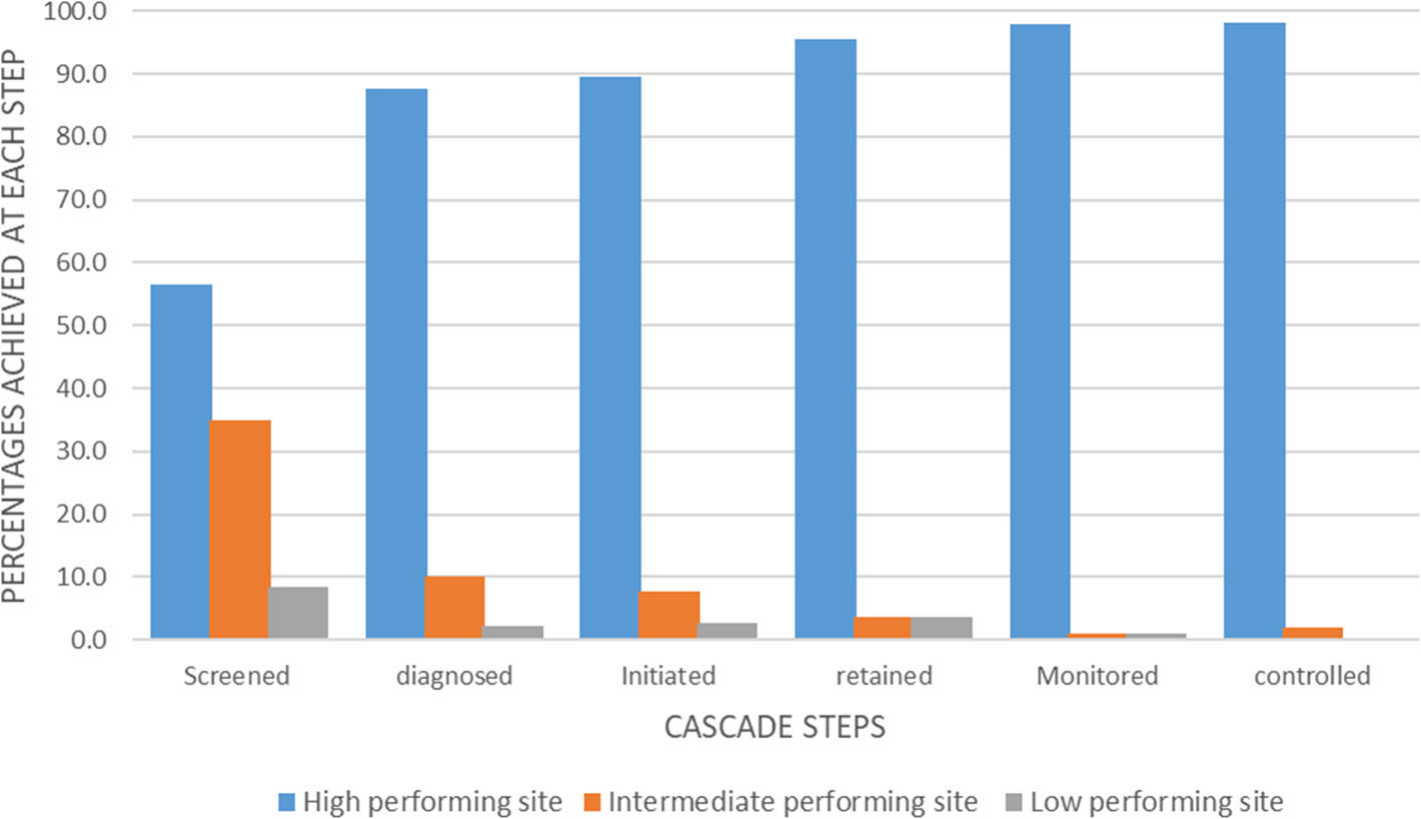
Differences in cascade outcomes across the three HIV clinics included in the study. The denominator for each cascade step is the achievement at the previous steps

The three clinics were our units of analysis. We used data from our recent retrospective cohort study [18] which assessed performance of each HIV clinic across HTN care cascade steps including screening, diagnosis, initiation of treatment, retention into care, monitoring, and BP control (This assessment is also highlighted in Fig. 2). Generally, the most significant care gaps were identified in screening and BP control [18]. Based on the cascade performance reported, we classified the three HIV clinics as high, intermediate, or low performing (Fig. 2).

#### Rating the CFIR constructs and interpretation

Ratings were performed to determine *valence*, which assesses whether the construct had a positive, neutral, or negative influence on implementation of integrated HTN/HIV care, and *strength* which is the degree of its influence. Positive influence indicated a facilitator of HTN/HIV integration while negative influence indicated a barrier. The coded text was then subjected to a rating process based on criteria shown in Table 1 [24]. We used a consensus process to assign a rating to each construct obtained from each HIV clinic based on the coded text. We based the rating of constructs on the level of agreement among study participants interviewed, strength of language, and use of concrete examples to emphasize responses (Table 1). After rating all constructs obtained from the three HIV clinics, we developed a matrix that listed the ratings for the CFIR construct for each of the clinics. We then focused our analysis on discerning patterns across the three HIV clinics. Using relative rating, but not absolute figures, we compared ratings across the HIV clinics and determined which constructs distinguished performance, either strongly or weakly or did not distinguish but influenced performance either negatively or positively. Barriers arose from constructs which distinguished performance strongly and had a negative influence on HTN/HIV service implementation at the lower performing HIV clinics. Facilitators arose from constructs which positively influenced HTN/HIV service provision at all the three HIV clinics. Complexity was “reverse rated” to be consistent with the other constructs, i.e., a positive sign denotes perception of simplicity, and a negative sign denotes complexity in implementation [22, 24, 25] (Table 3). We extracted specific quotations from the transcripts illustrating verbatim expressions of matters that appeared important. We followed the consolidated criteria for reporting qualitative research (COREQ) checklist in developing the manuscript [26]. We presented the findings to healthcare providers and patients who participated in the study at the three HIV clinics and the district.

**Table 1:** Criteria used to assign ratings to the CFIR constructs that influence screening and treatment of HTN in the HIV clinics

Rating	Criteria
− 2	The construct has a negative influence to HTN screening and treatment in the HIV clinic. An impeding influence in work processes and/or an impeding influence in implementation efforts. The majority of interviewees (at least two) described with explicit examples how the key or all aspects of a construct manifests itself in a negative way.
− 1	The construct has a negative influence to HTN screening and treatment in the HIV clinic, an impeding influence in work processes and/or an impeding influence in implementation efforts. Interviewees make general statements about the construct manifesting in a negative way but without concrete examples: • The construct is mentioned only in passing or at a high level without examples or evidence of actual, concrete descriptions of how that construct manifests • There is a mixed effect of different aspects of the construct but with a general overall negative effect • There is sufficient information to make an indirect inference about the generally negative influence and/or • Judged as weakly negative by the absence of the construct
0	A construct has neutral influence to HTN screening and treatment in the HIV clinic if: • It appears to have neutral effect (purely descriptive) or is only mentioned generically without valence • There is no evidence of positive or negative influence • Credible or reliable interviewees contradict each other • There are positive and negative influences at different levels in the organization that balance each other out, and/or different aspects of the construct that have positive influence while others have negative influence and overall, and the effect is neutral
+ 1	The construct is a positive influence to HTN screening and treatment in the HIV clinic, a facilitating influence in work processes, and/or a facilitating influence in implementation efforts. Interviewees make general statements about the construct manifesting in a positive way but without concrete • The construct is mentioned only in passing or at a high level without examples or evidence of actual, concrete descriptions of how that construct manifests; • There is a mixed effect of different aspects of the construct but with a general overall positive effect; and/or • There is sufficient information to make an indirect inference about the generally positive influence.
+ 2	The construct is a positive influence for HTN screening and treatment in the HIV clinic, a facilitating influence in work processes, and/or a facilitating influence in implementation efforts. The majority of interviewees (at least two) describe explicit examples of how the key or all aspects of a construct manifests itself in a positive way. Missing Interviewee(s) were not asked about the presence or influence of the construct; or if asked about a construct, their responses did not correspond to the intended construct and were instead coded to another construct. Interviewee(s) lack of knowledge about a construct does not necessarily indicate missing data and may instead indicate the absence of the construct.

## Results

TASO Tororo was the highest performing for all the six cascade steps. Mulanda HC IV was intermediate for screening, diagnosis, initiation of treatment, and control but lowest for retention and monitoring. Nagongera HC IV was the lowest for all the six cascade steps having achieved the same with Mulanda HC IV in retention and monitoring (Fig. 2).

The number and characteristics of participants for the interviews and focus group discussions are summarized in Table 2.

**Table 2:** Number and characteristics of participants involved in this study, by interview type and HIV clinic

Data collection methods	Mulanda health center IV	Nagongera health center IV	TASO Tororo	District Health Team (DHT)	Total participants	Mean age (SD)	Sex: male only freq (%)
Focus group discussion (FGDs) for patients	20	20	20	0	60	46.4 (± 7.2)	30 (50.0%)
In-depth Interviews (IDIs) for patient	4	4	4	0	12	47.2 (± 7.7)	06 (50.0%)
Key informant interviews (KIIs) for healthcare providers	Health facility manger (*n* = 1)	Health facility manger (*n* = 1)	Health facility manger (*n* = 1)	DHO (*n* = 1)	11	34.2 (± 7.6)	07 (63.6%)
	Lead nurse (*n* = 1)	Lead nurse (*n* = 1)	Lead nurse (*n* = 1)	0			
	Lead clinician (*n* = 1)	Lead clinician (*n* = 1)	Lead clinician (*n* = 2)	0			
**Total number of participants**	**27**	**27**	**28**	**1**	**83**		

Key: *SD* standard deviation, *Freq* frequency

Of the 39 CFIR constructs assessed, 17 were relevant to either barriers or facilitators to HTN/HIV integration. Barriers to HTN/HIV integrated care arose from the following six constructs: organizational incentives and rewards, available resources, access to knowledge and information, knowledge and beliefs about the intervention, self-efficacy, and planning. The barriers include lack of functional BP machines, inadequate supply of anti-hypertensive medicines, extra workload to providers for HTN services, inadequate knowledge about HTN care among PLHIV hence low demand, sub-optimal knowledge, skills and self-efficacy of healthcare providers to screen and treat HTN, and inadequate planning for integrated HTN/HIV services.

Relative advantage of offering HTN and HIV services in a one stop centre, simplicity (non-complex nature) of HTN/HIV integrated care, adaptability and compatibility of HTN/HIV care with the existing services in HIV clinics are the facilitators for HTN/HIV integration. The remaining 7 CFIR constructs were not significant regarding promoting or hindering HTN/HIV integration (Table 3). Detailed explanation of the barriers and facilitators are presented in Table 4. Below, we present the detailed results in the context of the five CFIR domains.

**Table 3:** Ratings assigned to CFIR construct by study site

	High performing site (C)	Intermediate performing site (B)	Low performing site (A)	Distinguishing constructs
**I. Intervention characteristics**				
Relative advantage	+ 2	+ 2	+ 2	Not
Adaptability	+ 2	+ 2	+ 2	Not
Complexity	+ 1	+ 1	+ 1	Not
**II. Outer setting**				
Patient needs and resources	− 1	− 2	− 2	Weakly
Peer pressure	− 2	− 2	− 2	Not
External policy and incentives	− 1	− 1	− 2	Weakly
**III. Inner setting**				
**Implementation climate**				
Compatibility	+ 2	+ 2	+ 2	Not
Relative Priority	− 1	− 2	− 2	Weakly
Organizational incentives and rewards	+ 1	− 2	− 2	Strongly
**Readiness for implementation**				
Available resources	+ 2	**−** 2	**−** 2	Strongly
Access to knowledge and information	+ 2	**−** 1	**−** 1	Strongly
**V1. Characteristics of individuals**				
Knowledge and beliefs about the intervention	1	1	**−** 2	Strongly
Self-efficacy	+ 1	**−** 2	**−** 2	Strongly
**V. Process of implementation**				
Planning	+ 1	**−** 2	**−** 2	Strongly
Executing	**−** 1	**−** 1	**−** 2	Weakly
Reflecting and evaluating	**−** 2	**−** 2	**−** 2	Not
Engaging: opinion leaders	**−** 1	**−** 1	**−** 1	Not

**Table 4:** Significant CFIR constructs and their related barriers or facilitators for integrated HTN/HIV care

CFIR Domain	CFIR Construct	Barrier or facilitator	Explanation of facilitators and barriers
**Intervention characteristics**	Relative advantage	Facilitator	Integrated HTN/HIV care saves time and costs on patient transport and improves patient retention. Patients receive both HTN and HIV care in the same clinic on the same appointment date.
	Adaptability	Facilitator	HTN/HIV integration fits within routine care in HIV clinics. HTN services can be tailored and refined to meet health needs of PLHIV.
	Complexity	Facilitator	Healthcare providers perceived provision of HTN care services in HIV clinics as straight forward and not complex.
**Inner setting**	**Implementation climate**		
	Compatibility	Facilitator	HTN/HIV integration was compatible and would fit within the existing workflows at the HIV clinics.
	Organizational incentives and rewards	Barrier	Lack of functional BP machines and medicines for HTN treatment in HIV clinics hinder HTN/HIV integration.
	**Readiness for implementation**		
	Available Resources	Barrier	Lack of functional BP machines, inadequate medicines to treat HTN, and extra work load to limited healthcare providers arising from offering HTN services hinder HTN service provision in HIV clinics.
	Access to knowledge and information	Barrier	Many PLHIV are not aware of HTN services at HIV clinics, hence low demand. Lack of training and continuing medical education for healthcare providers on HTN care hinders HTN/HIV integration.
**Characteristics of individuals**	Knowledge and beliefs about the intervention	Barrier	Some healthcare providers lacked knowledge and skills to screen and treat HTN in the HIV clinics.
	Self-efficacy	Barrier	Some healthcare providers lacked confidence in their own ability to screen and prescribe medicines for HTN in HIV clinics.
**Process of implementation**	Planning	Barrier	Inadequacies in preparation and planning for integrated HTN/HIV care: healthcare provider and patient orientation to integrated HTN/HIV care were generally suboptimal.

### Intervention characteristics

Healthcare providers at all three HIV clinics perceived HTN/HIV integration as a *relative advantage* and more effective compared to alternative modes of care for hypertensive PLHIV. Healthcare providers noted that integrated HTN/HIV saves time since patients received care for both HTN and HIV in the same clinic on the same appointment date, hence reducing costs on transport and improving retention.

*“*It is extremely important because if I were a patient having two chronic conditions, I would not want to spend my time going to a hospital for condition A and then go to another for condition B. HIV clinics should be a one-stop centre …*”* (KII, health facility manager HIV clinic C).

*Adaptability* was a facilitator for HTN/HIV integration since respondents at all the clinics perceived that HTN/HIV integration fits within their routine care provision. Healthcare providers stated: “HTN services can be tailored and refined to meet health needs of PLHIV.” *“Yes, hypertension management fits very well within our HIV care programs.”* (KII, health facility manager HIV clinic C).

*Complexity* was a facilitator for HTN/HIV integration. Across the HIV clinics, healthcare providers perceived provision of HTN care services as a task which was not complex and that activities for integrated HTN/HIV care were straightforward. A member of the DHT stated:

“For now, running the HIV clinics when providing care for opportunistic infections (OIs) and other NCDs isn’t difficult at these clinics.” (KII, DHT)**.**

### Outer setting

*Patient needs and resources* were not adequately prioritised and met by the prevailing HTN/HIV integrated care. Both healthcare providers and patients were in agreement that, to a large extent, HTN services at the HIV clinics were suboptimal to meeting the needs of PLHIV. Providers at lower performing HIV clinics added that HTN services were not adequately prioritized as evidenced by the low demand.

“Save for the good HIV care, it [HTN management] is almost not provided at this centre. … So, I seek treatment outside this centre.” (IDI 1, HIV clinic A).

“Patients are mainly interested in getting the HIV medicine [ART] refills. They do not demand for extra care unless their health has deteriorated”. (Lead nurse HIV clinic B).

“We also like to work on all patients’ conditions but we are sometimes limited by resources. Even now, not much is being done to support HTN integration.” (KII, DHO)*.*

Because the low implementing HIV clinics were not in a position to meet PLHIV’s needs for HTN management, hypertensive PLHIV were often referred to other health facilities, a strategy which HTN patients were not comfortable with.

“I was sent to Tororo general clinic (private for profit) to get checked and I was diagnosed with hypertension. I have the results with me but I’m always told to go back for care at the hospital.” (P2, FGD 2, HIV clinic A).

*External policy and incentives* was a weekly distinguishing construct that negatively influenced HTN/HIV integration. There were few external strategies for HTN/HIV integration through policies and guidelines. Although at the high performing HIV clinic, healthcare providers stated implementing the national HIV guidelines, and their emphasis was on the HIV component:

“Currently we are implementing the 2016 National guidelines for HIV/AIDS with emphasis on test and treat.” (Lead clinician HIV clinic C).

This construct was highlighted by healthcare providers at the low performing HIV clinic that they lacked comprehensive guidelines for HTN/HIV integration:

“We also lack specific standard operating procedures or guidelines to be followed in the HTN/HIV integration.” (KII, Lead clinician HIV clinic A)*.*

### Inner setting

Three sub-constructs under *implementation climate* were relevant to HTN/HIV integration. These were *compatibility, relative priority, and organization incentives and rewards* Compatibility was a facilitator for HTN/HIV integration. Healthcare providers perceived that HTN/HIV integration was compatible and would fit within the existing workflows at the HIV clinics. One of the healthcare providers noted:

“Yes, hypertension services do fit within our routine HIV care service provision.” (KII, lead clinician HIV clinic A)*.*

Relative priority was a weakly distinguishing construct which negatively influenced HTN/HIV integration. Although healthcare providers reported that HTN management would fit within the HIV clinic workflow, we found a *relatively lower priority* attached to HTN management by the healthcare providers compared to HIV care. Healthcare providers reported irregular provision of HTN services at the HIV clinic and providers from the low and intermediate performing facilities explicitly stated that:

“Basically, we provide the necessary HIV care services... and often patients are many; much work to do, so we prioritize HIV care”. (KII, lead clinician HIV clinic B).

“We often check the BP for patients with known hypertension and prescribe for them the medicines. However, those ones who are not yet known, we may check once in six months or when they complain with signs and symptoms suggestive of hypertension.” (KII, Lead clinician HIV clinic A).

Organization incentives and rewards was a barrier that strongly distinguished HTN/HIV integration. While healthcare providers at the high performing HIV clinic seemed to be motivated by the availability of equipment and other supplies to manage HTN, providers at the lower preforming HIV clinic s expressed the need to receive incentives like functional BP equipment and HTN treatment supplies or rewards to motivate their action for additional HTN services:

“Even the medicines and other equipment should be available. But we are still struggling to get better BP machines.” (KII, Lead clinician HIV clinic A).

“The HIV clinic does not receive special facilitation [payment] for managing hypertension cases.” (KII, Lead clinician HIV clinic B).

Readiness for implementation: the two sub-constructs: *available resources and access to knowledge and information* were barriers to HTN/HIV integration.

Lack of functional equipment for measuring BP, inadequate human resources, and medicines to manage HTN under the *available resources* construct were barriers to HTN/HIV integration. These resources were more available at the high compared to lower performing HIV clinics as noted by a healthcare provider in the high performing HIV clinic:

“Yes, I think we have adequate support. We have resources like drugs, equipment they are there, human resource.” *(KII,* Lead clinician HIV clinic C*)*.

On the contrary, low and intermediate performing HIV clinics reported lack of enough equipment especially functional BP machines. They also experience frequent stockouts of the medicines for HTN and the lack of specific funding towards HTN services:

“Yes. Most times we experience stockout of these HTN drugs, so we end up referring the patients.” (KII, facility manager HIV clinic B).

However, an increased number of hypertensive PLHIV at the HIV clinics with few healthcare providers would increase provider work load and patient waiting time as stated by a member of the DHT:

“Although …. when patients are many, it could be challenging for the few staff to offer care for the many tasks.”(KII, DHT)**.**

Access to information and knowledge was a barrier that strongly distinguished HTN/HIV integration between high and low performing clinics. At the high performing clinic, healthcare providers reported often having trainings including continuing medical education (CMEs) sessions on HTN.

“We always have CMEs on hypertension cases, we have had workshops and trainings, management of OIs and hypertension is part of it.” *(*KII, Lead clinician HIV clinic C).

On the other hand, healthcare providers at lower performing HIV clinics stated low *access to information and knowledge* about HTN care for PLHIV. They identified lack of trainings, few available trained staff at their HIV clinics, and poor learning environment as contributing factors.

“We have not had [trainings or capacity building sessions on hypertension management] for some time and that is the challenge that we have.” *(*KII, facility manager A*).*

In addition, many patients at low performing HIV clinics are not aware of HTN services at HIV clinic as mentioned by one healthcare provider:

“Most are not aware of other clinical service we provide including HTN management.” (Lead nurse HIV clinic B).

### Characteristics of individuals

*Knowledge and beliefs* about HTN/HIV integration was a strongly distinguishing construct and a barrier. At the high and intermediate HIV clinics, healthcare providers were acquainted with knowledge and skill to offer HTN services like BP measurement and prescription of medicine while at the low performance HIV clinic, and some of the healthcare providers lacked the skills to appropriately screen and treat HTN:

“I realized that health workers would report inconsistent BP measurements from patients. In case the triage says the blood pressure is high, I would measure again in the clinical room. Okay, there are some acceptable variations but there are those that are out of range. So it creates the need for more trainings.” (KII, lead clinician HIV clinic A)*.*

Self-efficacy, a strongly distinguishing construct, was a barrier to HTN/HIV integration at the low and intermediate HIV clinics. While healthcare providers at the high performing HIV clinic expressed confidence in their own ability to screen and treat HTN, some providers at the lower performing clinics expressed low self-efficacy to screen and prescribe medicines for HTN. One healthcare provider stated:

“Patients often present to us [with] different symptoms, in case you follow only these symptoms, you may think its pressure yet it’s not. I have sometimes used the BP machine, but because I don’t use it frequently, I don’t think I get exact measurements to conclude that one has pressure.” (KII Lead nurse HIV clinic B).

### Implementation process

Inadequacies in the planning of HTN/HIV integration especially when the program was being initiated was a barrier to the implementation. Healthcare providers reported that the implementation of HTN/HIV integration policy by MoH was suboptimal. This strongly distinguishing construct mainly affected the low performing clinics. Healthcare providers at these clinics reported insufficiencies in the preparation and support for HTN/HIV integration. The insufficiencies included lack of staff and professional training systems such as initial orientation to the new health guidelines. These healthcare providers noted that:

“The introduction of this program was not well communicated to the staff. A clear plan for HTN service provision should have been given to us and may be an official launch involving patients and we healthcare providers.” (KII, facility manager, HIV clinic A).

Although the planning and presentation of HTN/HIV integration were cited as insufficient, healthcare providers at the high implementing HIV clinic noted that they receive some support through their organization arrangements which has helped them to get acquainted with the HTN/HIV integration program.

“We didn’t have any capacity building at the start. However, as an organisation, we regularly have Continuous Medical Education (CME) on non-communicable diseases to boost the staffs’ knowledge. So we got some information on the suggested HTN/HIV integration strategies.” (KII, clinician, HIV clinic C)*.*

Execution of HTN/HIN integration was a weakly distinguishing construct as responses across the three clinics expressed suboptimal HTN services. Healthcare providers at the low performing HIV clinic mentioned:

“… we concentrate on the HIV care package ... We sometimes include hypertension, but not frequently.” (KII, lead clinician HIV clinic A).

In addition, healthcare providers at the low performing HIV clinic mentioned that they lacked comprehensive guidelines and standard operating procedure for HTN/HIV integration:

“We also lack specific standard operating procedures or documents to be followed in the HTN/HIV integration.” (KII, Lead clinician HIV clinic A)*.*

Besides, healthcare providers also reported insufficient support supervision from the health authorities including MoH, DHT, and implementing partners in relation to HTN management.

“No! We have not received any supervision at the HIV clinic, may be at the OPD [outpatient department]. Besides, when we are asked about the clinic, we are often asked about HIV care and supplies.” *(KII,* Lead nurse HIV clinic A*).*

### Feedback from participants when the result was shared

The DHO, healthcare providers, and patients with whom we shared results felt that the results were representative of their perceptions and perspectives regarding integrated HTN/HIV care. There was consensus among participants that there is need for support towards providing comprehensive HTN/HIV integrated care in HIV clinics. The key areas that needed support to improve include screening for HTN, access to anti-hypertensive medicines, and training of healthcare providers in HTN/HIV care.

Results for the three CFIR constructs which were non-significant regarding influencing HTN/HIV integration, namely, peer pressure, reflecting and evaluating, and engaging opinion leaders are presented in the appendix.

## Discussion

This study sought to evaluate factors that influence the integration of screening and treatment of HTN into the HIV program in Uganda, using the CFIR. We used valance rating to identify factors which distinguished performance for integrated HTN/HIV between the high and low performing HIV clinics. We found ten CFIR constructs which distinguished performance, and four of which were in the inner setting domain. Six of the constructs distinguished performance strongly while the remaining four weakly. All ten distinguishing constructs negatively influenced HTN/HIV integration at the low and intermediate performing HIV clinics as compared to the high performing clinic.

There were four constructs which positively influenced HTN/HIV integration at all the three HIV clinics but did not distinguish performance. These included relative advantage, adaptability, complexity of the intervention, and compatibility of HTN care with existing HIV services. We view these as the key facilitators for integrated HTN/HIV services in our setting. In agreement with the facilitators we identified for integrated HTN/HIV services, there is increasing demand for integrated rather than vertically oriented HTN and HIV services in low- and middle-income countries (LMIC) [5, 16, 17, 27–29]. Although some studies have assessed barriers and facilitators for HTN care in the HIV program, this is the first study in Africa to assess factors that influence HTN/HIV integration using the CFIR.

From the perspectives of both patients and providers across all the three clinics, integrated HTN/HIV care is seen to have a relative advantage as compared to vertically oriented programs. Providers agreed that having HTN/HIV integrated services will allow for improved patient-centred care. For example, patients would receive both HTN and HIV services instead of being referred to two different clinics. Furthermore, integration reduces duplication of services, is cost-effective, and efficient [5, 16, 29]. Our recent work also showed that the HIV cascade results were similar between patients with HIV alone and those with HIV and HTN who received integrated care [18]. Such evidence bolsters the demand for integrated HTN/HIV services from healthcare providers and patients that we see in the current study. Leveraging HIV programs for HTN care may even provide a spillover effect to the non-HIV population by increasing access to screening, treatment, and control of HTN [5, 27].

Adaptability was a positive influencer of integrated HTN/HIV services. Healthcare providers and leaders, especially in the high performing clinic, perceive that integrated HTN/HIV care can be adapted and tailored to fit the workflow of the HIV clinics to meet patient needs. HIV services were originally established as vertically oriented entities to address the emergency nature of HIV. Now that HIV has evolved into a chronic disease, largely due to these intensive, vertical efforts, leveraging successful HIV programs for NCD integration is highly recommended [16, 29]. A large clinical trial in Uganda that integrated HTN care into the HIV program showed that HTN control is better achieved in the HIV program as compared to the non-HIV population [30]. This is a true demonstration of the adaptability of integrated HTN/HIV service to the HIV program [30].

Most healthcare providers at all HIV clinics found HTN/HIV integration not to be complex, making it a facilitator. However, providers anticipated that as more dually affected patients are identified, the workload may become overwhelming if not met with a concomitant increase in staffing or resources. Task shifting could assist in mitigating the impact on providers [18]. In parallel with task shifting, as was and is done in the HIV context, programs must continue to strengthen the capacity of the existing clinical workforce to provide integrated care through re-education efforts, continuing medical education (CME), simplified evidence-based treatment protocols/algorithms, implementation guidelines, and physical and/or digital decision aides [5, 16]. Since access to information and knowledge was a barrier to HTN/HIV integration, capacity building for HTN and other NCDs in HIV should not only target clinicians but all cadres of staff so that the HTN/HIV services are supported by interdisciplinary teams for sustainability and efficient task shifting [5, 16].

Healthcare providers noted that HTN/HIV integration was compatible with the existing services in the HIV clinics and workflows. A project in Malawi that integrated screening and treatment of HTN into the HIV clinics demonstrated that HTN care was compatible with HIV services [5]. Indeed, as we demonstrated previously, the care cascades for both conditions can be well aligned [18].

All ten constructs which distinguished performance had a negative influence upon HTN/HIV integration. Design quality and packaging was a barrier to HTN/HIV integration. Healthcare providers from the lower performing clinics were not oriented on HTN/HIV integration, unlike those at the high performing HIV clinic. Additionally, Ugandan HIV programs will need to adopt the WHO target of 50% for HTN screening, treatment, retention in care, and control. Achieving this will require quality improvement efforts and integration of CVD indicators into routinely collected data at national, regional, district, and health facilities [27].

Patient needs and resources weakly distinguished performance for HTN integration. Key resources that hindered performance at the lower performing clinics included BP machines, access to medicines for HTN, and information on HTN/HIV integration. Fixed dose combinations of HTN medicines will reduce the pill burden of treating both HTN and HIV. Additionally, differentiated service delivery models and Chronic Care Models (CCM) for integrated HTN/HIV will strengthen treatment adherence and promote retention in care and patient centeredness [14, 15, 17, 29, 31].

Relative priority was a weak barrier to HTN/HIV integration. Healthcare providers mentioned that HTN screening and treatment among PLHIV are not prioritized. Providers mentioned that BP measurement at HIV clinic is only done for clients who are already confirmed to have HTN or those with symptoms but not all PLHIV. This non-prioritization of HTN services by providers leaves out many patients undiagnosed with HTN since majority have no symptoms especially in the early stages. This approach risks diagnosing patients very late with overt complications of HTN yet PLHIV are already in close contact with the healthcare system and present an opportunity for CVD screening, treatment, and control [29]. Due to suboptimal screening and treatment of HTN among PLHIV in Uganda, awareness of HTN and control remain below 20% [7–12, 18].

Providers at the high performing clinic were motivated by incentives and available resources including functional BP machines, access to medicines for HTN, and human resources unlike their counterparts at lower performing clinics who noted that they would derive motivation from the same incentives and resources. Our findings are in agreement with a recent mixed-methods study in Nigeria that assessed the capability, opportunity, and motivation for HTN/HIV integration found that physical opportunity in form of BP machines and medicines was suboptimal in most HIV clinics [28]. Strategies to improve access to medicines will require prioritization of availability and affordability of standardized selected core medications for HTN [5, 31].

Self-efficacy of healthcare providers to screen and treat HTN was low in the lower performing HIV clinics unlike in the high performing clinic. Additionally, clinicians at the lower performing clinics expressed lack of confidence in lower cadres of healthcare providers as far as screening for HTN was concerned. These findings are supported by prior studies that have found low levels of confidence and self-efficacy regarding HTN screening among multiple cadres of health workers [28, 32]. Strategies to address providers’ confidence will rely on improving knowledge and skills through cadre-appropriate training, mentorship, and education [5, 16].

Execution of HTN screening and treatment is generally suboptimal since it is at the discretion of the clinician especially at the lower performing HIV clinics. Despite the expectation that HIV clinics are to provide integrated HTN/HIV care according to WHO and MoH guidelines, evidence from this study shows that the guidelines are not generally implemented with fidelity. Suboptimal screening leads to low levels of awareness, treatment, retention, and control of HTN among PLHIV [7–12, 18]. Thus, there is a need to routinely provide HTN screening and treatment at HIV clinics, through use of standardized evidence-based treatment protocols, improved access to medicines, mentorship, target setting, improved systems for monitoring and evaluation, empowering patients, task shifting, differentiated service delivery, and community engagement [5, 31]. WHO and MoH guidelines should be revised to include the above strategies for effective HTN/HIV integration.

Participants in our study reported gaps in clinician documentation because providers record clinical data in patients’ personal books. These records leave the clinic with the patient following their visits. Enhanced systems for monitoring and evaluation for HTN in the HIV clinics are critically needed. This gap limits access to patient data and information for programmatic planning and continuous quality improvement. To close this gap, HTN care indicators should be integrated into the electronic medical records (EMR) system that is available and functional at HIV clinics [33].

## Limitations of the study

We acknowledge the paucity of contributions to the present analysis by the many patient participants interviewed for this study. Given there was no orientation of patients to integrated HTN/HIV care as part of planning, we received minimal responses and discussions by patients regarding the subject despite guidance by the interviewers. Few responses by patients about integrated HTN/HIV care may be an indicator of limited knowledge about hypertension in HIV. Patient education about hypertension services in HIV clinics is needed. Future implementation science research and analyses should place more emphasis on patients’ perspectives and perceptions on HTN/HIV integration.

## Conclusions

Using the CFIR framework, we have shown that there are modifiable barriers to integrating HTN services into the HIV clinic in the inner setting, outer setting, characteristics of individuals, and implementation process. Integrated HTN and HIV care is of great interest to both patients and healthcare providers. Improving access to HTN and other CVD care among PLHIV will require overcoming these barriers and capitalizing on the facilitators identified using a health system strengthening approach [5]. Findings from this study provide a springboard for designing contextually appropriate multicomponent interventions for HTN/HIV integration in Uganda and other LMICs. To further build the case for integrated HTN/HIV services, future research should determine the cost, cost effectiveness, and treatment outcomes for both HTN and HIV from integrated HTN/HIV services [16, 27].

## Supplementary information

**Additional file 1.** Results related to CFIR constructs that were less significant.

## Data Availability

The datasets used and/or analyzed during the current study are available from the corresponding author on reasonable request.

## Abbreviations

CFIR: Consolidated Framework for Implementation Research
HTN: Hypertension
PLHIV: Persons living with HIV
CVD: Cardiovascular disease
ART: Antiretroviral therapy
WHO: World Health Organization
MoH: Ministry of Health
FGD: Focus group discussion
KII: Key informant interview
IDI: In-depth interview
DHO: District Health Officer
DHT: District Health Team
LMICs: Low-and middle income countries
