# Supplementary material for: Exploring barriers and facilitators to integrated hypertension-HIV management in Ugandan HIV clinics using the Consolidated Framework for Implementation Research (CFIR)
Source: Implement Sci Commun. 2020 May 4;1:45. doi: 10.1186/s43058-020-00033-5 (PMC7427847; doi:10.1186/s43058-020-00033-5)
Supplement: Supplementary file 1 — Additional file 1. Results related to CFIR constructs that were less significant. [file 43058_2020_33_MOESM1_ESM.docx]

**Results on Consolidated framework for implementation research (CFIR)**

**Constructs that were non-significant**

**Peer pressure** was not a distinguishing construct and presented as a negative influencer to HTN/HIV integration. Health care providers were not aware of any HIV clinics implementing HTN/HIV integration to learn or derive competitive pressure from:

*“ I have not heard of any facility within or around the district [that is integrating HTN care]. Probably they are doing so, but there is no model health facility I can mention that we can learn from nor healthcare provider to share their experiences and best practices for this program as per now.” (KII,* lead clinician HIV clinic C*)*.

**Reflection and Evaluation,** was not a distinguishing construct but negatively influenced HTN/HIV integration. Healthcare providers reported that there was no formalized monitoring or evaluation process of HTN/HIV integrated services at the three HIV clinic. Besides, there were neither known set targets nor reporting/ feedback mechanisms and tools for HTN/HIV integration:

*“We rarely report on HTN care at HIV clinic. Since the HIV card has no provision for recording HTN data, we record on a separate paper which is added to the patient’s file.”* (KII, Lead nurse clinic B).

Another healthcare provider noted that:

*“We need to have HTN evaluation forms at these HIV clinics which we don’t do. For example, medical form 5, are often used up and out of stock, they are almost history at some facilities. Patients are encouraged to buy books …. We don’t have good record system to facilitate quick evaluation*.” (KII, DHO).

Some healthcare providers were concerned that quality improvement for HTN/HIV integration was not done for this program to be fully developed at their HIV clinics.

*“... we have not had any specific evaluation for this integration since it was communicated to us. Apart from the usual clinic reports that I send as a clinician, and I may inform them that we have a given number of hypertensive patients… but no specific reports made similar to the ones we make, say for TB.”* (KII, lead clinician HIV clinic A).

Under the **Engaging** construct, the **Opinion Leaders sub-construct was** non-distinguishing but negatively influenced HTN/HIV integration across the three sites. Key leaders at health facilities, district level and in some HIV implementing partners were not exhibiting commitment nor involvement in HTN/HIV integration. Besides, none of the HIV clinics had enthusiastic healthcare providers who were committed to coordinating or overseeing the implementation of HTN services.

*“We recently rolled out consolidated HIV management guidelines 2016, laid out how you can manage an HIV patient and they have also put emphasis on non-communicable diseases. …, the main issue is who is following up the implementation of care for non-communicable disease at these HIV clinics? This is because each health program and implementing partner concentrate on their targeted disease outcomes. So, you find that they can under look this integration of HTN care.”* (KII, DHO)**.**
